# Hereditary Angioedema-Associated Acute Pancreatitis in C1-Inhibitor Deficient and Normal C1-Inhibitor Patients: Case Reports and Literature Review

**DOI:** 10.3389/fmed.2019.00080

**Published:** 2019-04-17

**Authors:** Camila Lopes Veronez, Régis Albuquerque Campos, Rosemeire Navickas Constantino-Silva, Priscila Nicolicht, João Bosco Pesquero, Anete Sevciovic Grumach

**Affiliations:** ^1^Department of Biophysics, Universidade Federal de São Paulo, São Paulo, Brazil; ^2^Department of Clinical Immunology, Universidade Federal da Bahia, Salvador, Brazil; ^3^Department of Clinical Immunology, Faculdade de Medicina ABC, Santo André, Brazil

**Keywords:** hereditary angioedema, acute pancreatitis, abdominal swelling, C1-inhibitor deficiency, *F12* mutation

## Abstract

Abdominal pain due to intestinal swellings is one of the most common manifestations in hereditary angioedema (HAE). Bowel swellings can cause severe abdominal pain, nausea, vomiting, and diarrhea, which may lead to misdiagnosis of gastrointestinal disorders. In rare cases, HAE abdominal attacks can be accompanied by acute pancreatitis. Here, we report 3 patients with HAE and acute pancreatitis and present a literature review of similar cases. Patients with confirmed diagnosis of HAE secondary to C1-inhibitor (C1-INH) deficiency (*n* = 2) and HAE with normal C1-INH and *F12* mutation (F12-HAE) (*n* = 1) were included. Pancreatitis was diagnosed based on clinical symptoms and high lipase and amylase levels. Three HAE patients were diagnosed with acute pancreatitis based on increased amylase levels during severe abdominal swelling episodes. Two were previously diagnosed with HAE type I and one with F12-HAE. Pancreatitis was efficiently treated in two patients using Icatibant, with pain relief within hours. When conservatively treated, pancreatitis pain took longer time to resolve. Eighteen pancreatitis cases in HAE with C1-INH deficiency were previously reported and none in F12-HAE. Most patients (12/18) underwent invasive procedures and/or diagnostic methods. Although rare, severe abdominal HAE attacks could cause pancreatitis; HAE-specific treatments may be efficient for HAE-associated pancreatitis. HAE should be considered as a differential diagnosis of acute idiopathic pancreatitis. To our knowledge, this is the first report of HAE-associated pancreatitis in a F12-HAE patient treated with Icatibant.

## Background

Hereditary angioedema (HAE) is an autosomal dominant disease caused mostly by deleterious mutations in the gene encoding the C1-inhibitor (C1-INH) (C1-INH-HAE). It occurs in quantitative and functional C1-INH deficiency (type I) or functional C1-INH deficiency (type II) (OMIM #106100) (1). Another group of patients present with clinical characteristics of HAE with normal C1-INH (2). In these cases, HAE can be caused by specific mutations in *F12* gene (F12-HAE) (OMIM #610618) (2). Recently, HAE with normal C1-INH has been linked to mutations in genes encoding plasminogen (3) and angiopoietin 1 (4), however, there are still patients with unknown genetic cause.

The characteristic symptoms are nonpruritic and nonpitting swelling of submucosal or subcutaneous tissues involving the face, hands, feet, arms, legs, intestines, genitourinary tract, and upper airways, which can be life threatening (5). HAE symptoms generally last 2–5 days before resolving spontaneously without treatment; the most common trigger factors include emotional stress, infections, trauma, medical and surgical procedures, and estrogens (1, 5).

Abdominal pain is one of the most common manifestations of HAE (5, 6). Bowel swellings can cause severe abdominal pain, nausea, vomiting, and diarrhea. These symptoms may lead to misdiagnosis of gastrointestinal disorders; consequently, patients are frequently submitted to invasive procedures and unnecessary surgeries (5–7). In rare cases, HAE abdominal attacks can be accompanied by acute pancreatitis; this association is not fully understood and/or documented. Herein, we described 3 patients with HAE who presented with pancreatitis and abdominal attacks concomitantly and a review of other cases previously described in the literature.

This study was carried out in accordance with the recommendations of ethical standards of the 1964 Declaration of Helsinki. All subjects gave written informed consent in accordance with the Declaration of Helsinki. The protocol was approved by the Ethics Committee in Research of Universidade Federal de São Paulo (n°56522). Written informed consent for publication was obtained from all the participants of this case report.

## Case Presentation

### Case 1

A 21-year-old male patient presented to the emergency with severe abdominal pain of 7 h and 8 vomiting episodes. The first test showed normal amylase (93 U/L; normal 30-110 U/L) and slightly augmented lipase levels (332 U/L; normal 23-300 U/L). Analgesics were administered with partial improvement of pain. The second test performed 8 h after patient's admission revealed increase in levels of amylase to 292 U/L and lipase to 1,159 U/L, indicating acute pancreatitis. An increased volume of pancreatic tail, but no gallbladder, was observed through endoscopic retrograde cholangiopancreatography (ERCP) and magnetic resonance imaging (MRI) showed intestinal swelling. Two years before this episode of pancreatitis, he had been diagnosed with HAE type I, characterized by low C1-INH and C4 levels. The onset of HAE occurred at 1 year of age and consisted of facial edema triggered by trauma. Since then, he has been presenting with intermittent and irregular swelling episodes of the hands and feet, abdominal pain, and 3 episodes of upper airway edema. Due to HAE diagnosis, Icatibant (30 mg) was administered 19 h after admission, and the pain significantly reduced within 3 h. Amylase (69 U/L; normal 30–110 U/L) and lipase (165 U/L; normal 23–300 U/L) normalized 18 h after Icatibant injection and the patient was discharged the next day.

### Case 2

A 47-year-old female patient with C1-INH-HAE diagnosed 8 years earlier, presented to the emergency department with distended abdomen and severe abdominal pain lasting 24 h . The first test revealed increased amylase 210 U/L (normal 28–100 U/L), which considering a longer duration of abdominal pain indicated the development of pancreatitis. Since the hospital located in the North of Brazil had no vacancy, a single dose of Icatibant (30 mg) provided by the patient was administered and she was subsequently discharged. The next day, she presented with almost complete relief from the abdominal pain; in a total of 7 days, amylase and lipase reduced to normal levels. This patient has been presenting with recurrent angioedema attacks in the abdomen, face, limbs, and a few episodes in the upper airways, since she was 28-year-old. At that age, she underwent appendectomy and was misdiagnosed with Familial Mediterranean Fever. Only after 11 years, was she correctly diagnosed with C1-INH-HAE, confirmed using low C4 (6 mg/dL; normal 10–40 mg/dL) and C1-INH plasma levels (2 mg/dL; normal 19–40 mg/dL). She was treated with a prophylactic use of plasma-derived C1-INH and Icatibant during the attacks.

### Case 3

A 52-year-old female patient with F12-HAE (mutation p.Thr328Lys) had the onset of angioedema attacks at 16 years of age, during her first pregnancy. Symptoms were edema affecting the face, hands, and feet and abdominal pain. Currently, angioedema episodes occur monthly despite tranexamic acid prophylaxis (500 mg/day), mostly affecting gastrointestinal tract. Recently, one abdominal attack required 4 days of hospitalization. Pancreatitis was diagnosed using acute abdominal pain, high serum amylase levels (391 U/L; normal 25–125 U/L), and pathological signs at abdominal ultrasonography (US). She had normal leucocyte and platelet counts, total bilirubin, and aspartate aminotransferase. She was conservatively treated for pancreatitis due to the lack of the specific medication for HAE.

## Discussion

Acute pancreatitis is diagnosed if a patient presents with at least two of the following characteristics: (a) severe and persistent acute abdominal pain, (b) high serum lipase and/or amylase (3 times the normal upper limit), (c) and/or characteristic findings of pancreatitis on imaging tests (computed tomography, MRI, or US) (8). In addition, if the major and minor risk factors for acute pancreatitis is absent, like gallstones and alcohol misuse, and hereditary or drug-induced pancreatitis, other uncommon causes must be considered for an effective management (9).

The first report of pancreatitis associated with HAE is from 1986, which describes 3 C1-INH-HAE patients with recurrent abdominal attacks found to develop idiopathic chronic pancreatitis (10). In the sequence, more 12 independent cases have been reported, all are C1-INH-HAE (11–22) (Table 1). Although abdominal attacks are quite frequent among the different types of HAE, monitoring pancreatic enzymes is not routinely performed. Aksoy et al. (20) described a male patient diagnosed with HAE type I only after presenting with 4–5 episodes of abdominal pain and pancreatitis, despite that swelling of the face, lips, and tongue had occurred in the first attack (20). In another report, a 6-year-old boy with acute pancreatitis was diagnosed with HAE only after 10 hospitalizations in a period of 18 months. Within this period, this patient underwent cholecystectomy, however, no gallstones were found (16).

**Table 1:** Case reports of pancreatitis in HAE patients.

**References**	**N°/gender**	**Age,y**	**HAE type**	**Amylase (IU/L) (normal range)**	**Lipase (IU/L) (normal range)**	**Treatment for attacks / Prophylactic treatment**	**Effect**	**Invasive procedures**
Case 1	1/M	21	I	292 (30–110)	1,159 (23–300)	Icatibant (30 mg)	Significant abdominal pain relief within 3 h.	ERCP.
Case 2	1/F	47	I	210 (28–100)	ND	Icatibant (30 mg)	Alleviation of symptoms in hour and resolution in 48 h.	Appendectomy.
Case 3	1/F	52	F12-HAE	391 (25-125)	ND	Withholding food intake, intravenous hydration, analgesics.	Improvement in a few days.	ND
Brickman et al. (10)	3/ ND	ND	I	ND	ND	ND	ND	Abdominal surgery in 2 patients. Marsupialization.
Cutler et al. (11)	1/F	44	I	95–100; 150 (< 85)	400 (0–210)	Danazol, diphenhydramine, corticotropin, propranolol, hydrocortisone.	Unsuccessful	Esophagogastroduodenoscopy, ERCP, appendectomy, tonsillectomy.
Matesic et al. (12)	1/M	40	I	1,117 (0–130)	11,485 (0–95)	Intravenous hydration, antibiotic prophylaxis, analgesics, nasogastric decompression, proton pump inhibitor. Prophylactic danazol (200 mg/day).	Marked improvement in 3 days. Resolution in 7 days.	Esophagogastroduodenoscopy, ERCP.
Chung et al. (22)	1/F	51	ND	369	1,735	Withholding food intake, intravenous hydration, analgesics. Prophylactic danazol.	Improvement in a few days.	ND
Majoni and Smith (13)	1/M	43	I	340 (35–110)	ND	C1-INH concentrate. Discharged of danazol due to renal impairment.	Abdominal pain resolved in 24 h. Normalization of amylase in 3 days. Resolution in 7 days.	Tonsillectomy, appendectomy.
Marín Garcia et al. (14)	1/M	49	II	445 (25–115)	2,342 (114–286)	ND Prophylactic tranexamic acid (20 mg/day) after discharge.	ND	Tonsillectomy, endoscopy.
Cancian et al. (15)	1/F	32	I	ND	ND	C1-INH concentrate (18 U/kg).	Alleviation of symptoms in 30 min and resolution in 16 h. Normalization of amylase in 24 h.	ND
Czaller et al. (16)	1/F	29	I	2,615 (28–100 U/L)	1,452 (13–60 U/L)	C1-INH concentrate (500 IU) twice, methimazole sodium, drotaverine. Prophylactic danazol.	Alleviation of symptoms in 4 h and resolution in 24 h.	Appendectomy.
Ben Maamer et al. (17)	1/F	73	I	869 U/L	1,235 U/L	Intravenous fluids, antibiotic prophylaxis, analgesics, nasogastric decompression, proton pump inhibitor. Prophylactic danazol (200 mg/day).	Alleviation of symptoms in 5 days. Discharge after 14 days.	Esophagogastroduodenoscopy.
Berger et al. (18)	1/M	6	I	1,440 (10–100 U/L)	180 (16–65 U/L)	Fluids, papaverin, low-fat diet (before HAE diagnostic). C1-INH concentrate (after HAE diagnostic).	Alleviation of symptoms, normalization of amylase and lipase levels. Disappearance of symptoms within minutes.	Cholecystectomy.
Loudin et al. (19)	1/F	56	I	ND	663 IU/L	C1-INH concentrate (20 mg/kg).	Disappearance of symptoms after 30 min. Lipase decreased to 142 IU/L.	Cholecystectomy, endoscopic ultrasound.
Aksoy et al. (20)	1/M	55	I	ND	ND	ND Prophylactic danazol (200 mg/day).	ND Free of symptoms (3-month follow-up).	Laparotomy, partial omentectomy.
Hirose et al. (21)	1/ND	ND	I or II	ND	ND	ND	ND	ND

*ERCP, endoscopic retrograde cholangiopancreatography; ND, not described; F, female; M, male*.

Not only is pancreatitis underdiagnosed, HAE patients frequently undergo invasive or improper diagnostic methods and treatments (5, 23), especially when they present with isolated or predominantly abdominal swellings. Patient 2 underwent appendectomy and spent years relying on a wrong diagnosis. In Case 1, the patient was submitted to an ERCP during the pancreatitis investigation. Likewise, in the other 15 published cases, 7 invasive diagnostic procedures and 12 surgeries possibly related to HAE had been done (Table 1). These include 3 appendectomies and 3 tonsillectomies, 2 cholecystectomies, 2 non-specified abdominal surgeries, 1 laparotomy, 1 omentectomy, and 1 marsupialization (Table 1). In a prospective study, one Japanese patient presented with acute abdomen with intestinal edema; acute pancreatitis was newly diagnosed with C1-INH-HAE (21).

Patients with HAE and normal C1-INH present with similar clinical manifestations as HAE-C1-INH ones. Some authors described facial edema as the most frequent event among F12-HAE (24), nevertheless, we observed that more than 60% of these patients had recurrent abdominal swellings (6). Here, we report the first patient with pancreatitis associated with HAE attack.

During HAE attacks, the obstruction of pancreatic duct or ampulla of Vater can result from duodenal edema, the most probable cause of pancreatitis in this situation. Pancreatic edema itself is not reported in HAE. Aksoy et al. (20) reported mild pancreatic duct dilation and side duct ectasia using magnetic resonance cholangiopancreatography during an HAE-associated pancreatitis episode, supporting the hypothesis that obstruction of pancreatic duct is responsible for pancreatitis in HAE (20); however, in other case reports, pancreatic duct presented normal (12, 17, 19). The obstruction of pancreatic duct causes the blockage of pancreatic secretion, leading to zymogen granules fusion with lysosomes, trypsinogen activation, and autodigestive injury of acinar cells, stimulating inflammatory response (9).

Since angioedema attacks resolve spontaneously in 2–5 days, HAE-associated acute pancreatitis should also disappear within a few days. This was observed in Case 3, and in the reviewed cases in which pancreatitis was treated conservatively (11, 12, 17, 18). However, we cannot establish if the episode described in Case 3 is a pancreatitis associated with an HAE swelling, or a single acute pancreatitis, since no specific medication was administered. Indeed, as well as HAE abdominal attacks are frequently misdiagnosed and mistreated, a comprehensive evaluation of abdominal pain in HAE patients unsuccessfully treated with specific medication should be performed.

When specific treatment for acute HAE attacks was administered (C1-INH replacement), the alleviation and resolution of symptoms was remarkably faster, within hours to a maximum of 1 day (13, 15, 16, 18, 19). C1-INH administration was beneficial in different experimental animal models of pancreatitis (25–27), but ineffective in others (28). Although the benefits of C1-INH therapy are questionable in general pancreatitis, the cases here reviewed indicate good efficacy in HAE-associated pancreatitis.

For the first time, we observed improvement of clinical symptoms caused by abdominal pain and pancreatitis with the administration of Icatibant, a specific antagonist of bradykinin B2 receptor, in Cases 1 and 2. B2 receptor antagonists were shown to diminish pain associated to pancreatitis (29), to reduce pancreatic plasma extravasation, and pancreatic neutrophil influx (30), and to reduce serum amylase and lipase (31) in different pancreatitis-induced models. Although a direct activation of B2 receptors in pancreatitis pathogenesis can be speculated (by both circulating or local bradykinin released by tissue kallikrein), the role of the kinin system in pancreatitis is still unknown.

To our knowledge, other treatments available for acute HAE attacks have not reported for pancreatitis yet. In addition to patient from Case 1, six of the reported patients were under prophylactic treatment with danazol, an attenuated androgen which increases the production of hepatic C1-INH. On the other side, danazol can also be associated with pancreatitis development (9, 32), but in this case, pancreatitis should not resolve spontaneously. Moreover, elevated liver enzymes, a common sign of steroid injury, had been described in only one of the patients presenting pancreatitis and taking danazol (12). One of our patients (case 3) presented with high gamma-glutamyl transferase and slightly high alanine aminotransferase, but she was under long-term prophylaxis with tranexamic acid, a plasmin inhibitor, and not steroids. Moreover, we could not find a causative association between danazol prophylaxis and HAE-associated pancreatitis in any of the 18 cases described.

In conclusion, we present here the first description of HAE-associated acute pancreatitis in a F12-HAE patient and the first cases treated with icatibant. Although HAE attacks are rarely associated with pancreatitis, our findings and the review of literature suggest to investigate HAE in idiopathic pancreatitis. Likewise, pancreatitis signs should be searched for in severe abdominal attacks in HAE patients to avoid further complications. The efficient response to C1-INH replacement and B2 receptor antagonist (Icatibant) indicates that specific drugs for HAE attacks can be useful options for HAE-associated pancreatitis in addition to the conservative treatment.

## Ethics Statement

This study was carried out in accordance with the recommendations of ethical standards of the 1964 Declaration of Helsinki with written informed consent from all subjects. The protocol was approved by the Ethics Committee in Research of Universidade Federal de São Paulo (n°56522).

## Author Contributions

CV, RC-S, and PN performed the analysis and reviewed the literature. CV conceptualized and drafted the manuscript. RAC and AG performed the data collection instruments, and coordinated data collection. AG and JP conceptualized the study, coordinated and supervised data collection. All the authors drafted, reviewed and approved the manuscript.

### Conflict of Interest Statement

AG and CV received grants from Shire International GmbH (Investigator Initiated Research–IST-BRA-000778 and IIR-BRA-002232). The remaining authors declare that the research was conducted in the absence of any commercial or financial relationships that could be construed as a potential conflict of interest.
